# The relative fitness of drug-resistant *Mycobacterium tuberculosis*: a modelling study of household transmission in Peru

**DOI:** 10.1098/rsif.2018.0025

**Published:** 2018-06-27

**Authors:** Gwenan M. Knight, Mirko Zimic, Sebastian Funk, Robert H. Gilman, Jon S. Friedland, Louis Grandjean

**Affiliations:** 1 National Institute of Health Research Health Protection Research Unit in Healthcare Associated Infections and Antimicrobial Resistance, Imperial College London, Commonwealth Building, Hammersmith Campus, Du Cane Road, London W12 0NN, UK; 2 Infectious Diseases and Immunity, Imperial College London, Commonwealth Building, Hammersmith Campus, Du Cane Road, London W12 0NN, UK; 3 Centre for the Mathematical Modelling of Infectious Diseases, Infectious Disease Epidemiology, London School of Hygiene and Tropical Medicine, London WC1E 7HT, UK; 4 TB Centre, London School of Hygiene and Tropical Medicine, London WC1E 7HT, UK; 5 Laboratorio de Bioinformática y Biología Molecular, Facultad de Ciencias, Universidad Peruana Cayetano Heredia, 31 Avenue Honorio Delgado 430, Distrito de Lima, Peru; 6 Johns Hopkins Bloomberg School of Public Health, 615 N Wolfe Street, Baltimore, MD 21205, USA; 7 Wellcome Trust Imperial College Centre for Global Health, St Mary's Hospital Campus, Imperial College London, Praed Street, London, W2 1NY, UK

**Keywords:** drug-resistance, tuberculosis, fitness, mathematical modelling

## Abstract

The relative fitness of drug-resistant versus susceptible bacteria in an environment dictates resistance prevalence. Estimates for the relative fitness of resistant *Mycobacterium tuberculosis* (*Mtb*) strains are highly heterogeneous and mostly derived from *in vitro* experiments. Measuring fitness in the field allows us to determine how the environment influences the spread of resistance. We designed a household structured, stochastic mathematical model to estimate the fitness costs associated with multidrug resistance (MDR) carriage in *Mtb* in Lima, Peru during 2010–2013. By fitting the model to data from a large prospective cohort study of TB disease in household contacts, we estimated the fitness, relative to susceptible strains with a fitness of 1, of MDR-*Mtb* to be 0.32 (95% credible interval: 0.15–0.62) or 0.38 (0.24–0.61), if only transmission or progression to disease, respectively, was affected. The relative fitness of MDR-*Mtb* increased to 0.56 (0.42–0.72) when the fitness cost influenced both transmission and progression to disease equally. We found the average relative fitness of MDR-*Mtb* circulating within households in Lima, Peru during 2010–2013 to be significantly lower than concurrent susceptible *Mtb*. If these fitness levels do not change, then existing TB control programmes are likely to keep MDR-TB prevalence at current levels in Lima, Peru.

## 1. Background

*Mycobacterium tuberculosis* (*Mtb*) is a highly prevalent bacterium, thought to infect just under a quarter of the world's population [1]. Treatment of tuberculosis (TB) disease is not simple and drug-susceptible tuberculosis (DS-TB) requires a multiple-drug regimen taken for at least six months [2]. Multidrug-resistant tuberculosis (MDR-TB) treatment regimens are significantly longer, cause serious side effects and are very expensive [3]. While currently 5% of all TB cases globally are estimated to be MDR-TB [2], predicting the future burden of DS- and MDR-TB is essential for TB control programmes.

One key parameter that determines the future prevalence of drug-resistant TB is the relative fitness of drug-resistant *Mtb* strains when compared with drug-susceptible *Mtb* strains [4–7]. Fitness is a complex, environment-dependent trait that can be defined as the ability of a pathogen to survive, reproduce, be transmitted and cause secondary cases of disease. These abilities are affected by multiple environmental factors such as a host's genetics, the current TB treatment regimen and other risk factors for transmission, which are all time-varying. The importance of this parameter has been highlighted by several mathematical models which show how even small changes in its value can predict widely varying future levels of MDR-TB burden [4–6,8,9]. Thus, gaining environment-dependent, accurate estimates of fitness is of critical importance.

Within *Mtb,* it has been shown that the appearance of drug resistance (MDR) mutations affects fitness [10–12]. These previous studies have shown that resistant *Mtb* is, usually, less fit than susceptible *Mtb* under a range of fitness definitions: either by demonstrating a lower growth rate *in vitro* (e.g. [13]), less progression to disease after inoculation in guinea pigs (e.g. [14]) or a lower chance of causing secondary cases of disease (e.g. [12,15]). The latter definition is important for epidemiological predictions of burden, while the first provides the potential underlying biological cause. The epidemiological fitness of an *Mtb* strain can be split into an ability to (1) cause secondary infections (transmission) and (2) cause subsequent active disease (progression). For example, resistant *Mtb* may be transmitted equally, but subsequent disease rates in those infected may be lower or less severe. For *Mtb* this split is especially pertinent due to the importance of the latent, non-infectious stage of disease.

Also highly important for *Mtb* is the spatial location of transmission [16]. Few studies have considered the critical influence of household structure on transmission of *Mtb*. To our knowledge, no studies have considered the spread of drug-resistant tuberculosis in the context of a household-structured stochastic mathematical model.

The difference in definitions of fitness and corresponding experimental data makes translation from data analysis to predictive mathematical modelling difficult. Here, we tackle this problem by fitting a mathematical model to a detailed dataset on the transmission of *Mtb* strains collected in a large cohort study of households undertaken in Lima, Peru between 2010 and 2013 [17]. We derive estimates of fitness in this specific setting with different fitness definitions (either effects on transmission and/or progression to disease) and test the robustness of these estimates under a range of assumptions. These parameters will allow for better predictions of future MDR-TB levels and an improved understanding of MDR-TB spread.

## 2. Material and methods

### 2.1. Data

The details of the underlying study and participants can be found in [17]. Briefly, 213 and 487 households were recruited with an index case of diagnosed MDR- or DS-TB, respectively, during 2010–2013. Households were followed up for variable periods of time up to a maximum of 3 years (electronic supplementary material, figure S1). During the study households were visited every six months, and household contacts were monitored for TB disease. It was found that 35/1055 (3.32%, 95% CI [2.32, 0.4.58]), of the MDR-TB contacts, and 114/2356 (4.84%, 95% CI [4.01, 5.78]), of the DS-TB contacts developed TB disease, suggesting that DS-TB has higher fitness. There were no significant differences between cohorts by HIV status, age, gender or household size [17].

The specific data used to calibrate the model were (i) the incidence of MDR-TB and (ii) DS-TB in households with an index DS-TB case and (iii) the incidence of MDR-TB and (iv) DS-TB in households with an index MDR-TB case (table 1). The percentages of incident cases with resistance profiles matching the index was used to multiply the incidence levels accordingly.

**Table 1.** Fitted parameters with description, prior distributions, any differences by model structure and data used for fitting. All parameters are fitted to the TB incidence date from the household (HH) study [17]. The three models have different assumptions around the effect of decreased fitness, with *f* varying to be *f*_1_ (affects transmission rate) or *f*_2_ (affects progression to disease rate) (figure 1).

symbol	parameter description	prior distribution	Model 1	Model 2	Model 3	data
foi*_s_*	external force of infection of DS-TB	uniform [0; 0:5]		/		DS-TB incidence in MDR-TB index HH: 4264 [3916, 4338]
foi*_r_*	external force of infection of MDR-TB	uniform [0, 0:3]		/		MDR-TB incidence in DS-TB index HH: 87 [13, 435]
*f* (*f*_1_, *f*_2_)	relative fitness of MDR-TB strains compared to DS-TB strains, which have a fitness of 1	uniform [0, 1]	*0* < *f*_1_ < 1 *f*_2_ = 1	*f*_1_ = 1 0 < *f*_2_ < 1	*f*_1_ = *f*_2_ 0 < *f*_1_ < 1	MDR-TB incidence in MDR-TB index HH: 2112 [1646, 2358]
*β*_s_	*per capita* transmission rate of DS-TB within households	uniform [90, 140]		/		DS-TB incidence in DS-TB index HH: 4264 [3916, 4338]
*β*_r_	*per capita* transmission rate of MDR-TB within households	calculated from other fitted parameters: *β*_r_ = *f*_1_*β*_s_				

### 2.2. Model structure

**Figure 1.**
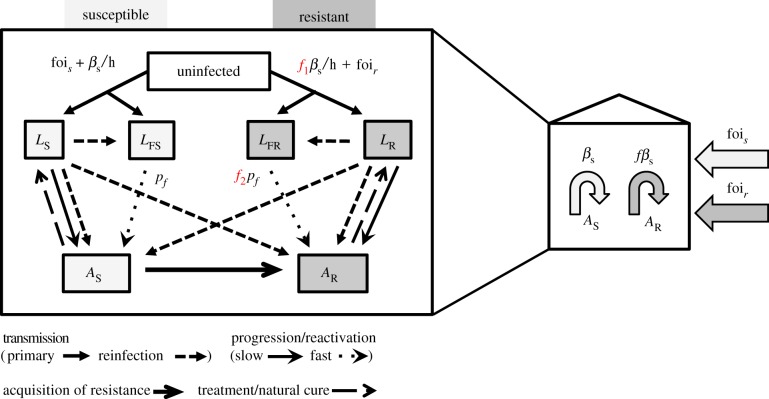
A standard natural history, transmission model for two strains (susceptible and resistant) of *Mtb* was used (diagram on the left). Uninfected people become infected at a rate dependent on the number of active cases (dynamic transmission). Once infected, the majority of people (85%) are assumed to enter a latent slow (*L*_S_/*L*_R_) state. The remainder enter a rapid progression (latent fast, *L*_FS_/*L*_FR_) state which has a higher rate of progression to active disease (*A*_S_/*A*_R_). Resistance mutations are acquired during active disease. Those with active disease recover to the latent slow state via treatment or natural cure. The fitness cost to resistance is assumed to affect the rate of transmission (*f*_1_) or the rate at which those latently infected with MDR-TB progress to active disease (*f*_2_). Only the effect on primary transmission of *f*_1_ is highlighted here, but reinfection is also affected. *f*_1_ and *f*_2_ are set at 1 or allowed to vary between 0 and 1 in the three separate models: *f*_1_ in Model 1, *f*_2_ in Model 2, and both *f*_1_ and *f*_2_ in Model 3. The four estimated parameters (shown in the diagram on the right) were rates of internal transmission (*β*_s_, *f*) and the external forces of infection (foi_*s*_, foi_*r*_). (Online version in colour.)

The mathematical model was a standard two-strain dynamic TB model (figure 1), with transmission modelled at the level of the household. A Gillespie stochastic simulation algorithm in *R* [18] was developed using the R package ‘GillespieSSA’ [19]. Using a stochastic transmission model was important as the model was implemented independently in households where the small populations mean stochastic effects are highly important. The size of the household varied, with household size sample from data on the distribution in the original household cohort study [17]. We assumed that saturation of transmission could occur and hence scaled our transmission rate by the size of the household (number of people), assuming households have the same ventilation level (or at least that this did not vary by index case *Mtb* resistance status) and within-household homogeneous mixing [20]. This assumption of frequency-dependent transmission means that, in households with more people, household members are assumed to have lower individual chance of infection from an active disease case than in smaller households, due to decreased exposure. This has been observed for another airborne pathogen, influenza [21] and was explored in sensitivity analysis where we also considered density-dependent transmission. All natural history parameters were taken from the literature and are listed in table 2, and the dynamics explained in the legend to figure 1.

Four parameters were estimated from the data (table 1 and figure 1): (1) the *per capita* transmission rate of DS-TB within households (*β*_s_), (2) the relative fitness of MDR-*Mtb* strains versus DS-*Mtb* strains (*f*) expressed as an effect on transmission or progression or both, and the external (to households) force of infection (foi) of (3) DS-TB foi_*s*_ and (4) MDR-TB foi_*r*_.

Our main outcome was the impact of resistance on transmission rates, but we also explored the impact on an approximate effective reproduction number (see the electronic supplementary material).

### 2.3. Three model formulations

Resistant strains were allowed to have an equal or lower fitness relative to susceptible strains. The mechanisms behind this reduction were estimated to affect two different rates: the transmission rate, the rate of progression to disease, or both (figure 1). We assumed that the fitness of the resistant strains could not rise above that of susceptible strains due to the data from the household cohort [17]. Model 1 (transmission fitness cost model) assumed that fitness costs directly affected the number of secondary infections by reducing the transmission parameter for MDR-*Mtb* (0 < *f*_1_ < 1, *f*_2_ = 1, figure 1). This is the standard assumption for the effect of resistance on fitness for transmission dynamic models of *Mtb* [6,9,38] and other pathogens [39]. Model 2 (progression fitness cost model) assumed that although MDR-TB transmission occurred at the same rate as DS-TB, there is a fitness cost to progression to disease (*f*_1_ = 1, 0 < *f*_2_ < 1, figure 1). Model 3 assumed that there was a fitness cost to both transmission and progression, and that the cost was the same for both processes (0 < *f*_1_ = *f*_2_ < 1, figure 1). We could not explore a model with fitness affecting both processes at differing levels as we did not have data on levels of infection. Without these data, a model with high transmission fitness cost but low progression cost would be equally as likely as a model with a low transmission fitness cost but a high progression cost and hence would be uninformative. Note that fixing either *f*_1_ or *f*_2_ equal to one is the same as ignoring this parameter altogether and leaving the multiplied rate at its background level as they are both scalar constant parameters with no units.

### 2.4. Model simulation

The model initially sampled 700 household sizes (with replacement) from the exact distribution of household sizes in the trial [22]. Initial numbers of people with latent infection were sampled from a normal distribution generated by data from the literature [2,40] (see the electronic supplementary material). The model was then simulated for 10 years with an MCMC sampled set of the four unknown parameters (pre-study period), capturing transmission within the household prior to enrolment in the household study. A random time point from this 10-year period in which there was at least one active case with the same sensitivity as the initial case in the household (i.e. DS-TB or MDR-TB) was taken to be the time the household entered the study and the active index case was detected. This allows for simulation of changes in latency in the household and provides initial conditions dependent upon each parameter sample.

The above randomly chosen time point of entry to the study was taken to be the initial conditions for the simulation of the model that was fitted to the household study [17] (study period). The same values of the four unknown parameters were used as in the pre-study period and the simulation time for each household was randomly sampled (with replacement) from the exact distribution of follow-up times in the study (electronic supplementary material, figure S1). The only parameter that changed, to match the altered patient care in the study, was the case detection rate which increased for the study period from the WHO estimates to a screen occurring every six months (table 2).

**Table 2.** Parameter values with description and baseline values. All prior distributions were uniform.

symbol	parameter description	baseline value	prior distribution	notes and references
*Nr*	number of households with MDR-TB index case	213	/	[22]
*Ns*	number of households with DS-TB index case	487	/	[22]
*h*	household size	2–15	/	[17]
*p*	proportion of (re-)infected individuals who progress to the ‘latent fast’ state	0.15	0.08–0.25	[23–25]
*χ*	protection from developing active TB upon reinfection	0.35	0.25–0.45	[23,26–29]
*ϕ*	rate of reactivation among those latently infected per year	1.13 × 10^−4^	1–3×10^−4^	[23,26,27,29–31]
*ɛ*	probability of acquiring new drug resistance during treatment	0.008	0.005–0.01	[32]
*d*	proportion of new active cases which directly become infectious	0.5	0.25–0.75	[23,29,33,34]
*μ*	background death rate	1/77 = 0.013	0.012–0.014	inverse of average life expectancy in Peru [35]
*μ_A_*	additional death rate of those actively infected and infectious per year	0.26	0.2–0.4	[23]
*N*	annual rate of natural cure for TB cases (returns to latent state)	0.2	0.15–0.25	[23]
*ω_s_*	proportion of DS-TB active cases detected and treated per year	0.8; 2	0.5–0.95	for 2012 [2] for pre-study; in study: screen every 6 months
*ω_r_*	proportion of MDR-TB active cases detected and treated per year	0.64; 2	0.2–0.9	79% of the above 80% (*ω_s_*) found that received DST in 2012 [36]; in study: screen every 6 months
(*1 – k_s_*)	proportion of DS-TB active cases started on treatment that are successfully cured	0.74	0.5–0.9	[36,37] (for midpoint of study)
(*1 – k_r_*)	proportion of MDR-TB active cases started on treatment that are successfully cured	0.6	0.2–0.9	for 2012 [2]
*p_f_*	progression rate of latent fast individuals to active disease	0.2	0.1–0.9	duration of fast latency period of 5 years [27]

The TB incidence from the model was calculated by determining the total number of new cases of active TB in all 700 households over the follow-up time, and dividing this by the total number of follow-up years in these households. The total number of follow-up years was a product of the number of household members and the follow-up time for the household, taking into account any deaths over this time. We assumed that none left the households other than by death (natural or due to TB). Time with active disease was included in the follow-up. For a detailed overview of the process see electronic supplementary material, figure S2.

### 2.5. Model fitting

Approximate Bayesian computation (ABC) was paired with Markov chain Monte Carlo (MCMC) methods to estimate the four unknown parameters [41]. All other parameters were kept fixed at their baseline value (table 2). The summary statistic used was the TB incidence from the model falling within the 95% CI for all four TB incidence measures from the data. Uniform priors were assumed for all four parameters (table 1).

To estimate the standard deviation required for the MCMC for the four unknown parameters, Latin hypercube sampling (LHS) from the prior ranges was initially used (Stage A, electronic supplementary material). The empirical standard deviation from the accepted fits was then used as the proposal distribution of a Metropolis–Hastings MCMC sampler (Stage B), used to estimate posterior probabilities of the parameters.

We used the generated trajectories to consider the probability of remaining free of tuberculosis from the model output and compared the trends to the data (fig. 2 from [17]).

### 2.6. Scenario analysis

A scenario analysis was used to explore the sensitivity of Model 1 results to key natural history parameters. Firstly, we changed the initial proportion of the population latently infected with MDR-*Mtb* from 2% to 10%.

A full sensitivity analysis of the parameters kept fixed in the model fits was not possible due to limitations imposed by computation time. Instead, to determine which further scenarios to explore, we determined the parameters most correlated with TB incidence in our model, and hence likely to have the biggest impact on our model fit and parameter estimates. To determine these parameters, we used LHS to choose 10 000 parameter sets from (uniform) prior distributions for all parameters (table 2). We then ran Model 1 with these 10 000 parameter sets and determined the parameters that were statistically significantly correlated with any of the four TB incidence outputs (Kendall correlation, *p* < 0.01). These parameters were then used to design two scenarios—one with a combination of these parameters at their prior values which gave highest TB incidence and the combination which gave the lowest TB incidence.

We also increased our 10-year initial run-in period for the population to 30 years and explored the impact on the estimates. Furthermore, we explored removing the assumption of saturating household transmission (*per capita* transmission rate was then not dependent on household size, i.e. density-dependent transmission).

All code is available online [42].

## 3. Results

### 3.1. Fit to the data

**Figure 2.**
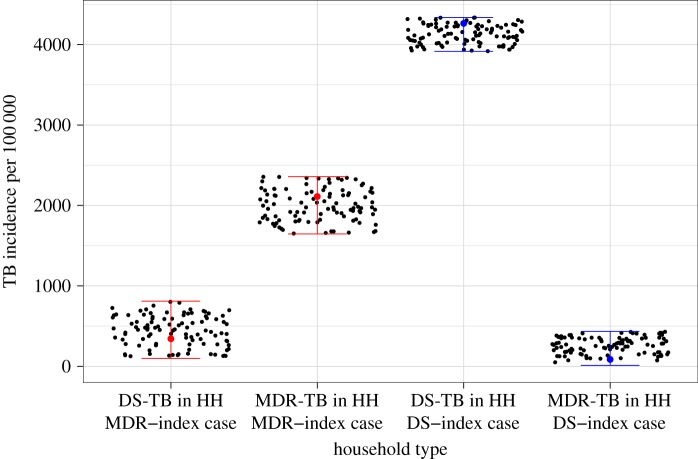
Model fits. Black dots represent the Model 1 output that matches with the data shown in ranges for each type of household (HH). See electronic supplementary material, figures S3 and S4 for equivalent plots for Model 2 and 3. (Online version in colour.)

Model structures 1–3 could all replicate the data from the household study (figure 2). The MCMC trace and density plots of the posterior distributions are shown in the electronic supplementary material.

### 3.2. Parameter estimates

**Figure 3.**
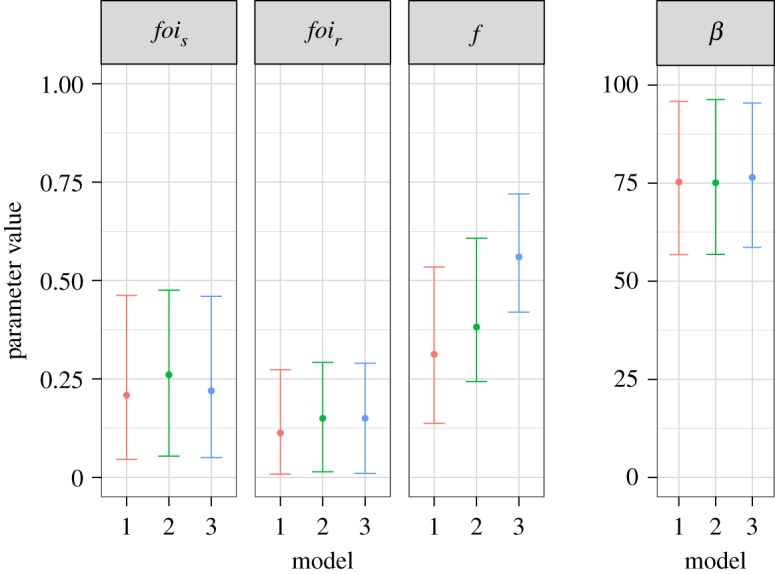
Fitted parameters from each model. The units for the *y*-axis of the corresponding plots are: for the external forces of infection (*foi_s_* and *foi_r_*) proportion infected per year, for the relative fitness (*f*) there are no units and for the *per capita* transmission rate (beta) the units are effective contact rate per year. Model 1 assumes a transmission cost to resistance, Model 2 a disease progression cost and Model 3 assumes an effect on both. (Online version in colour.)

The estimates of the external foi for DS- and MDR-TB were similar across the three models (table 3 and figure 3). The *per capita* transmission rate of DS-TB within households was also similar across the three models. The relative fitness of MDR-*Mtb* was similar for Model 1 and 2, but increased in Model 3, as might be expected as in this third model the reduction in fitness is applied to two rates. For Model 1, that is assuming a resistance phenotype affects transmission, the relative fitness of MDR-*Mtb* was estimated to be 0.32 (median, 95% CI: 0.15–0.62) versus DS-*Mtb* with a fitness of 1. In Model 2, where a resistance phenotype affected disease progression, a similar relative fitness was estimated: 0.38 (0.24–0.61). If both rates were affected, then the relative fitness of MDR-*Mtb* was estimated to be 0.56 (0.42–0.72) (table 3 and figure 3).

**Table 3.** Parameter estimates for the median and 95% credible intervals of the four unknown parameters from 50 000 MCMC iterations with a burn-in of 10 000 iterations. The fitness cost to resistance is assumed to affect transmission in Model 1, progression to active disease in Model 2, and both transmission and progression in Model 3.

Model	foi*_s_*	foi*_r_*	*β*	*F*
1	0.22 (0.03–0.49)	0.10 (0.01–0.26)	74.70 (54.80–97.60)	0.32 (0.15–0.62)
2	0.26 (0.05–0.48)	0.15 (0.01–0.29)	75.08 (56.85–96.30)	0.38 (0.24–0.61)
3	0.22 (0.05–0.46)	0.15 (0.01–0.29)	76.45 (58.60–95.42)	0.56 (0.42–0.72)

Comparing the external foi for DS- versus MDR-TB, we found that the ratio of the two was around 0.5 (median estimate 0.45/0.58/0.68 from the three models). This single value for the external foi represents a complex set of processes (contact patterns, length of infectiousness etc.) and hence cannot be used to determine relative fitness. However, the ratio is in the range that supports our estimates of the relative fitness from the internal household model. The ratio of an approximate effective reproduction number for MDR- and DS-TB also supported our main results (see the electronic supplementary material).

### 3.3. Probability of remaining free from tuberculosis

We explored the probability of remaining free from tuberculosis as was presented from the original study (fig. 2 in [17]). By comparison we had highly similar dynamics to the study (see electronic supplementary material, figure S5).

### 3.4. Scenario analysis

Our five scenarios gave very similar estimates for the relative fitness of MDR-*Mtb* (a range of medians from 0.27–0.34; electronic supplementary material). This suggests that the estimates of relative fitness are robust to: increasing the initial proportion of households that were initially infected with latent MDR-*Mtb* from 2% to 10% (in the pre-study), setting TB incidence to high or low levels (see the electronic supplementary material for parameter details), extending the initial run-in period from 10 to 30 years; or removing the saturation of transmission within households.

## 4. Discussion

Our results suggest that the average relative fitness of MDR-*Mtb* strains circulating in households in Lima, Peru in 2010–2013 was substantially lower than that of drug-susceptible strains (approx. 40–70% reduction). When a resistance phenotype was assumed to affect both transmission and progression to disease rates, then the relative fitness of MDR-TB strains was approximately 60%.

The strengths of this study are that we were able to fit a stochastic household-level model to detailed location-specific data, accounting for accurate distributions of both household size and study follow-up time. We were also able to differentiate between internal and external transmission, matching the resistance typing data from the household study [17]. Moreover, our transmission rate estimates account for the longer infectiousness of MDR-TB cases (due to delays in diagnosis and treatment initiation etc.). This model and its MCMC fitting algorithm can be applied to other settings and then used as the basis for predictions of future levels of DS- and MDR-TB. In particular, this novel way of estimating fitness costs, by fitting dynamic transmission models to resistance-specific incidence data could be used for other TB-prevalent settings or for other bacteria. Furthermore, the estimates given can be directly translated into dynamic transmission models for prediction while previous estimates, for example, of differences in growth rates have less clear epidemiological translations.

Our modelling analysis is limited by the assumption of homogeneity of both hosts and strains. The characteristics of the DS- and MDR-TB contacts under consideration in the underlying household study were highly similar [17]. Thus, as our estimate is of a relative fitness, we believe that including host differences in our model may have had little effect on our relative results. Strain heterogeneities, however, mean that our result is (potentially) an average across many different drug-resistant strains. It is known that differences in resistance and compensatory mutation combinations result in a diversity in fitness across strains [13]. This diversity is highly important for predictions of MDR-TB levels in the future [43]. Our estimate must therefore be taken as a population average in Lima, at a certain time and indicative of the mean fitness rather than an indicator of the range of potential fitness in the population. If one highly fit MDR-TB strain were to emerge (or were already present), then future prevalence predictions based on our (mean) estimate could be an underestimate. We fitted the model to data with confidence intervals that were derived without fully accounting for the dependency of infection between household members. Improving methods for robust approximation of parameters from mechanistic models that take full account of such dependencies is an important active area of research [44], and will improve future studies of this kind.

Our Model 1, where a transmission effect is assumed, is the most similar to previous models of MDR-TB transmission [6,9,38]. Reductions in transmission could arise from many factors including differences in location of infection (pulmonary versus non-pulmonary), a different interaction with the basic immune system or different aerosolization levels. However, our MDR-TB fitness predictions are at the lower end of the range seen previously [10]. This may reflect the situation in Peru where there is a strong tuberculosis control infrastructure with a well-developed MDR-TB treatment programme and a growing economy. These two factors may have combined to limit the spread of MDR-TB and hence prevent the adaptation of MDR-TB to a higher fitness. At the bacterial level, compensatory fitness mutations that could influence the ability of drug-resistant *Mtb* strains to spread may not have emerged or not been allowed to spread. Calibrating the model to other settings would help clarify this issue. Alternatively, it may be that our estimates are providing, for the first time, a better direct translation of fitness from epidemiological data to a transmission model parametrization.

There is a paucity of evidence for whether differences in TB disease prevalence in general are due to infection or progression to disease [45]. In particular, for resistant strains it is unclear where the effect of becoming resistant should be applied in the natural history of tuberculosis infection. Both Snider and Teixeira [46,47] demonstrated similar levels of tuberculin skin test (TST) conversion among MDR- and DS-TB household contacts but lower levels of disease in contacts of those with MDR-TB. This was also seen in a recent study in children [48], while a higher prevalence of TST positivity was found in household contacts of MDR-TB patients than contacts of newly diagnosed TB patients in Viet Nam [49]. This evidence combines to suggest that the fitness cost to resistance, if any, was to be observed on the progression to disease. We make this assumption in our Model 2, where the hypothesis is that those with active TB disease, whether due to resistant or susceptible bacteria, have a similar bacterial load and hence ability to transmit successfully. However, once successfully established in a new host, resistant bacteria may be less able to combat the immune system and establish a disease state. This has been assumed in a previous model of HIV and MDR-TB interaction [50].

Previous models have assumed that resistant strains could become more fit (i.e. have a relative fitness greater than 1), while we capped the relative fitness of the resistant strains at 1, due to the data from previous studies and the literature [13,51]. Our posterior parameter distributions for the estimated relative fitness parameter (reflected in the 95% CI for *f*; see the electronic supplementary material) suggest that this is a valid assumption for the resistant strains circulating at this time in Lima. Importantly, all our estimates are of ‘relative’ fitness, and therefore should be robust to changes in natural history assumptions as these would affect both drug-susceptible and -resistant strain transmission.

Future work will include adding details on host and strain heterogeneity to the model. Data collection of strain heterogeneity along with active contact tracing and an understanding of where and from whom transmission occurs would drastically improve our understanding of fitness and hence improve estimates of future MDR-TB levels. Exploring the external infection methods and potential changes in this foi over time (i.e. making it dynamic as in [52]) would allow for models that can predict levels of MDR-TB in Lima. Future predictive transmission modelling using our relative fitness estimates are likely to suggest that if treatment objectives are maintained and this fitness measure remains constant, MDR-TB prevalence will remain under control in Lima in the short term.

In conclusion, we find the fitness cost of MDR in *Mtb* in Lima, Peru to be substantial. Importantly, this paper provides direct transmission model estimates, using a novel method, of the relative fitness levels of drug-resistant *Mtb* strains. If these fitness levels do not change, then the existing TB control programmes are likely to keep MDR-TB prevalence at their current levels in Lima, Peru. These estimates now need to be gained for *Mtb* in other settings and the values used in models to explore future global burden.

## Supplementary Material

Supplementary Material

## Supplementary Material

Follow up time

## Data accessibility

The datasets supporting this article have been uploaded as part of the electronic supplementary material. All code used is available on GitHub and Zenodo [42].
