# Supplementary material for: The relative fitness of drug-resistant Mycobacterium tuberculosis: a modelling study of household transmission in Peru
Source: J R Soc Interface. 2018 Jun 27;15(143):20180025. doi: 10.1098/rsif.2018.0025 (PMC6030636; doi:10.1098/rsif.2018.0025)
Supplement: Supplementary Material [file rsif20180025supp1.pdf]

Supporting Information for: The relative fitness of drug resistant *Mycobacterium tuberculosis*: a modelling study of household transmission in Peru

Contents

1 Time of follow-up in study **2**

2 Detailed overview of simulation **3**

3 Models 2 & 3: Fit to data **4**

4 Probability of remaining free from tuberculosis **6**

5 Trace and density plots for each unknown parameter for main models **7**

6 Result: scenario analysis: Fit to data **10**

7 Trace and density plots for each unknown parameter for scenario analysis **15**

8 Scenario analysis results **20**

9 Effective Reproduction Number estimates **22**

1 Time of follow-up in study

Households were followed-up for variable lengths of time in the original household study (Supplementary Figure 1). The raw data is included in the Electronic Supplementary Material.

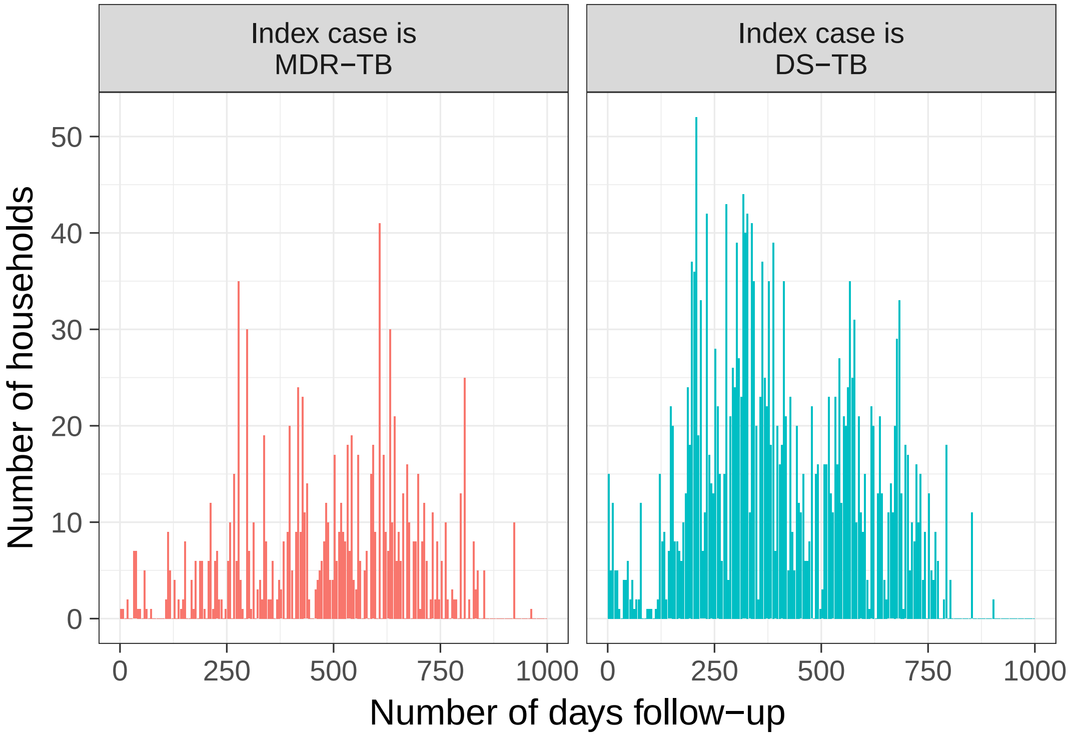

Figure 1: Distribution of follow-up times for households with an index case that was MDR-TB (left) or DS-TB (right).

2 Detailed overview of simulation

A detailed overview of all the stages used in the simulation are provided in Figure 2.

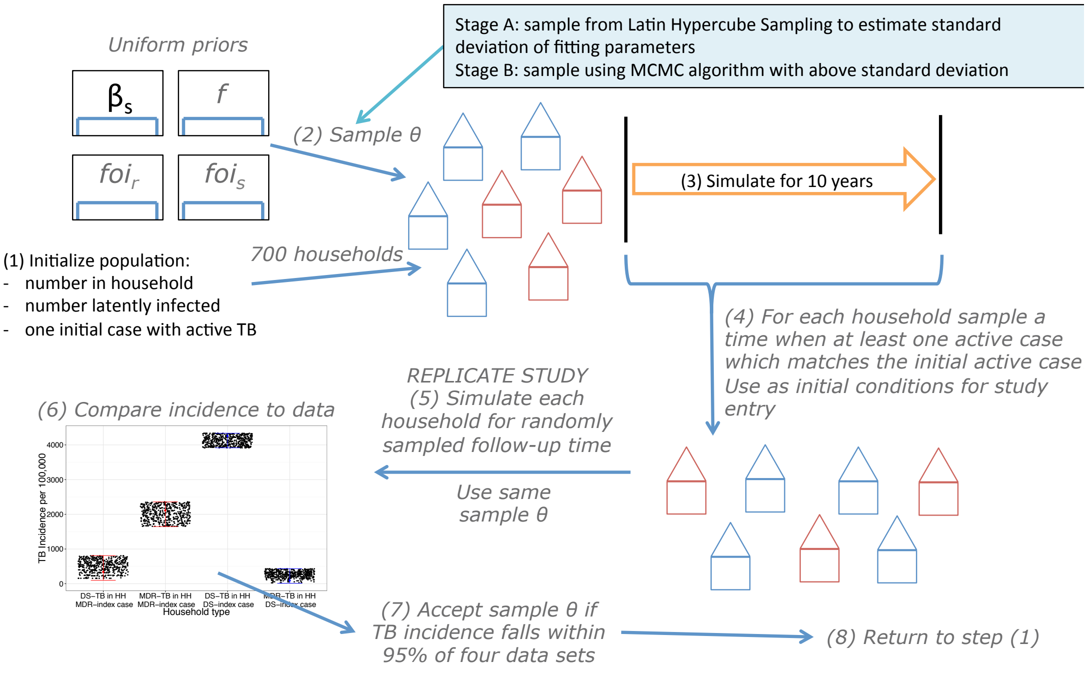

Figure 2: A pictorial representation of the simulation stages.

The model initially sampled 700 household sizes from the distribution of household sizes in the trial.[1] 213 of these had an initial MDR-TB case, 487 an initial DS-TB case. Tuberculin skin test (TST) prevalence surveys across Lima have found 52% (95% CI: 48-57%) to be infected with Mtb.[2] Hence, the number of cases initially latently infected was sampled from a normal distribution with mean 0.5 and standard deviation of 0.1. Informed by the TB prevalence in Lima, it was assumed that initially, 98% of these latent infections were with DS-TB strains, 2% with MDR-TB strains in all households.[2] This proportion was varied in scenario analysis. Random sampling from a binomial distribution, with this 98% DS-TB, determined the distribution of latent DS-TB and MDR-TB cases across the 700 households. The proportion of latent cases that were ”latently fast” cases (Figure 1) was taken to be 3% to reflect that although the proportion of new infections that are fast latent is 15%, over time these will change state more rapidly than latent slow.

3 Models 2 & 3: Fit to data

Model 1-3 structures could all replicate the data from the household study as shown in Figure 2 in the main paper and Supplementary Figures 3 & 4.

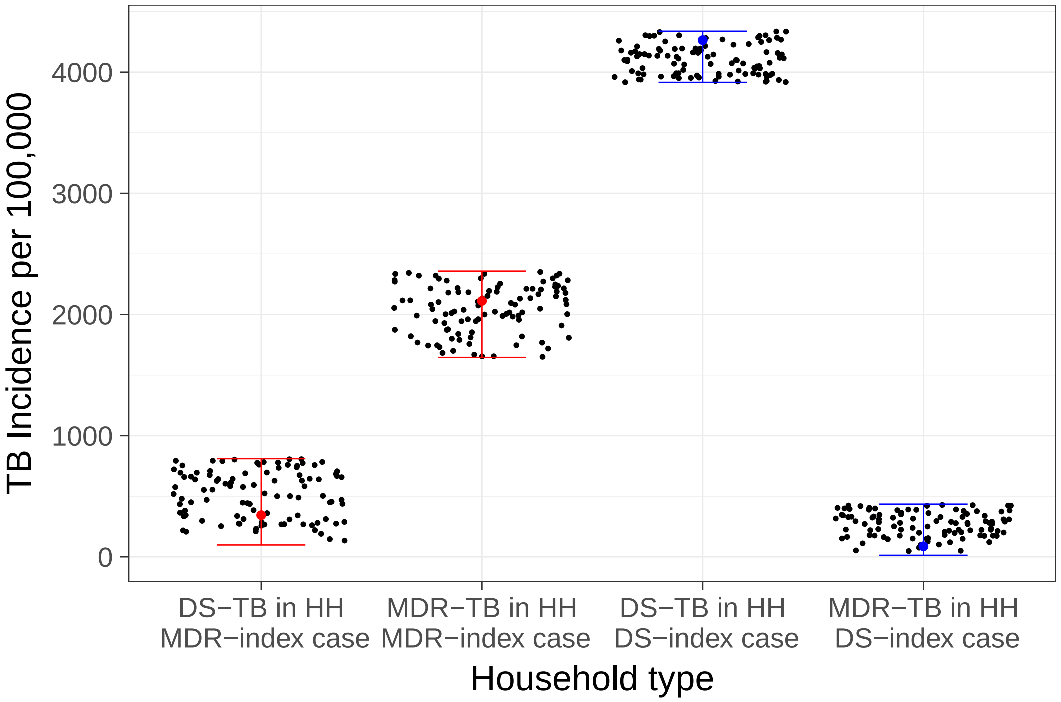

Figure 3: 100 example model fits. Black dots represent Model 2 output that matches to data shown in coloured ranges for each type of household (HH).

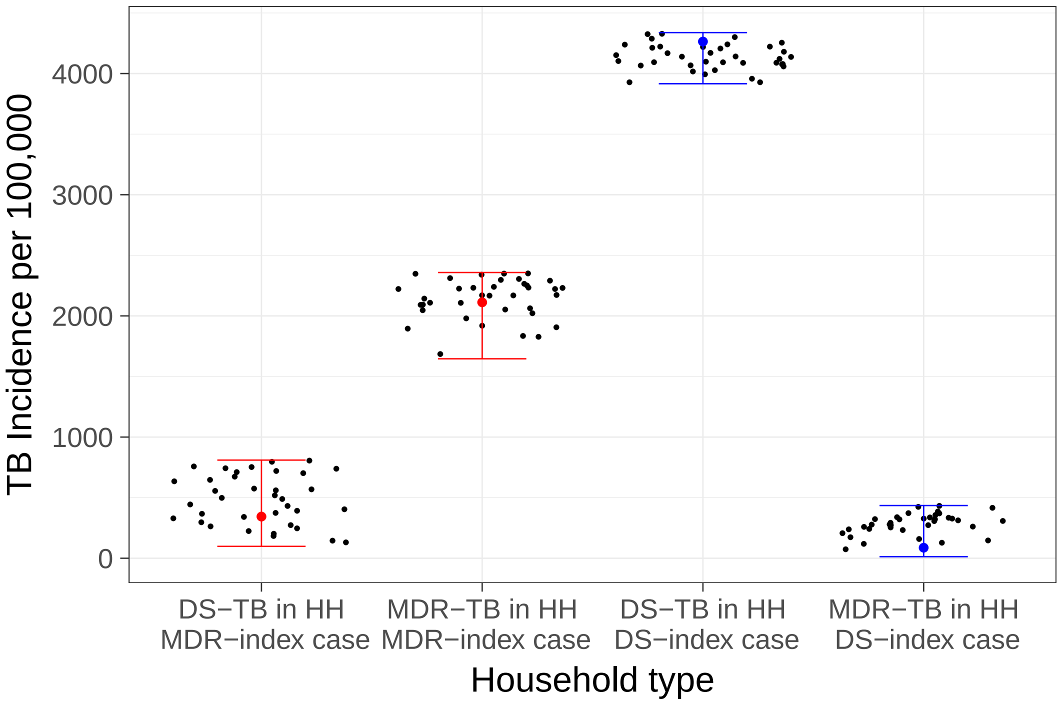

Figure 4: 100 example model fits. Black dots represent Model 3 output that matches to data shown in coloured ranges for each type of household (HH).

4 Probability of remaining free from tuberculosis

We compared the probability of remaining free from TB in our model to that presented in the original study (Figure 2 in the original paper by Grandjean et al.[1]). We had highly similar dynamics to those in the main study (Figure 5).

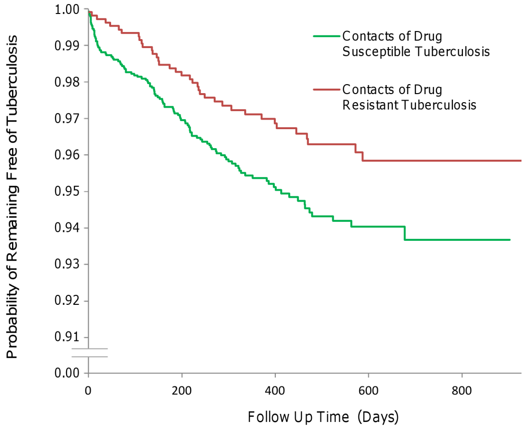

(a) Data from study

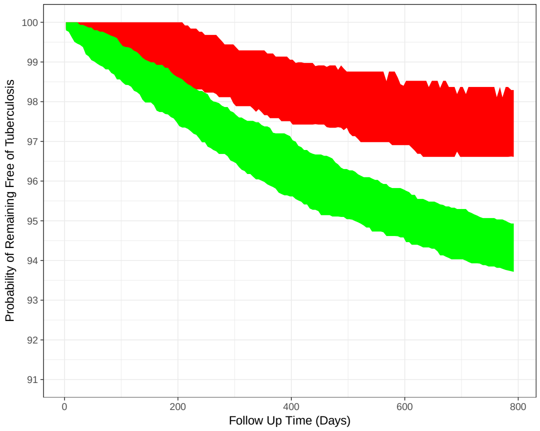

(b) Model 1

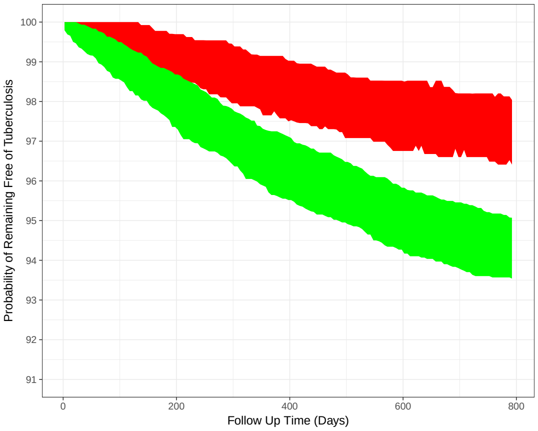

(c) Model 2

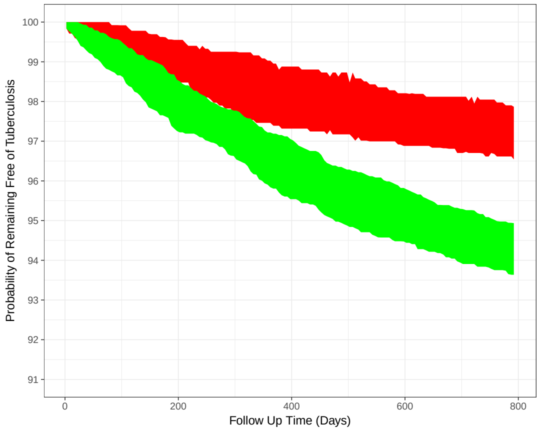

(d) Model 3

Figure 5: Probability of remaining free from tuberculosis for study[1] (a) and three model structures (b-d).

5 Trace and density plots for each unknown parameter for main models

The trace and density for each unknown parameter, from the three models are shown in Supplementary Figures 6-8.

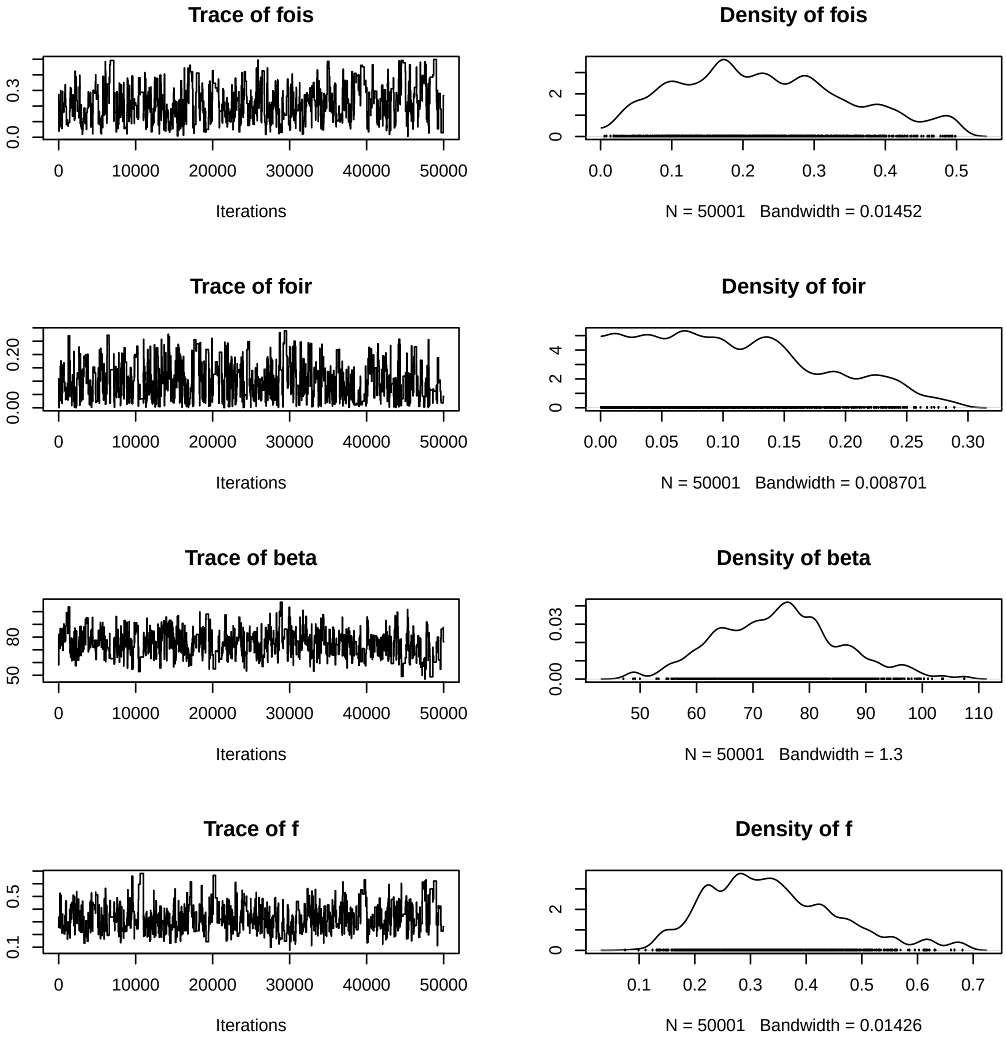

Figure 6: Trace and density plots for Model 1

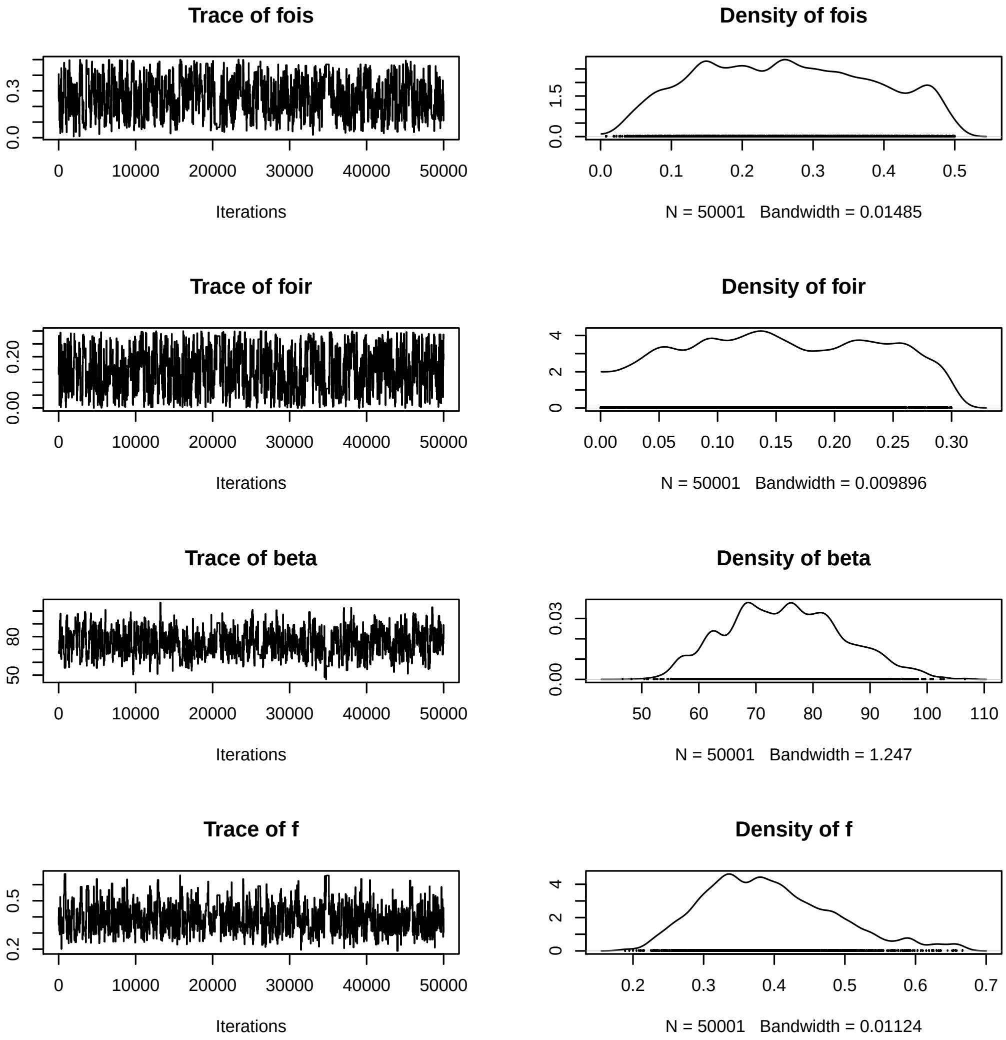

Figure 7: Trace and density plots for Model 2

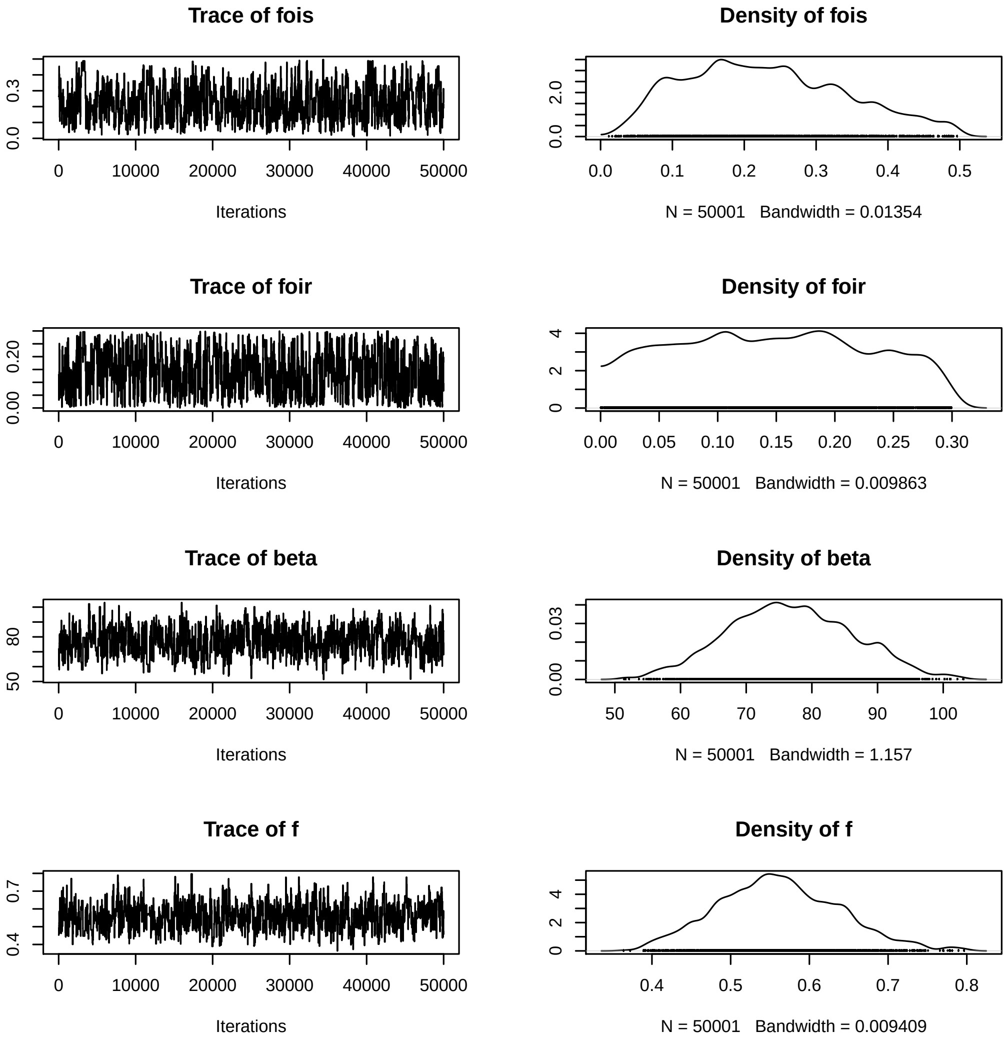

Figure 8: Trace and density plots for Model 3

6 Result: scenario analysis: Fit to data

Scenario analysis used the structure from Model 1 with altered parameters. All four could replicate the data from the household study as shown in Supplementary Figures 9 - 11.

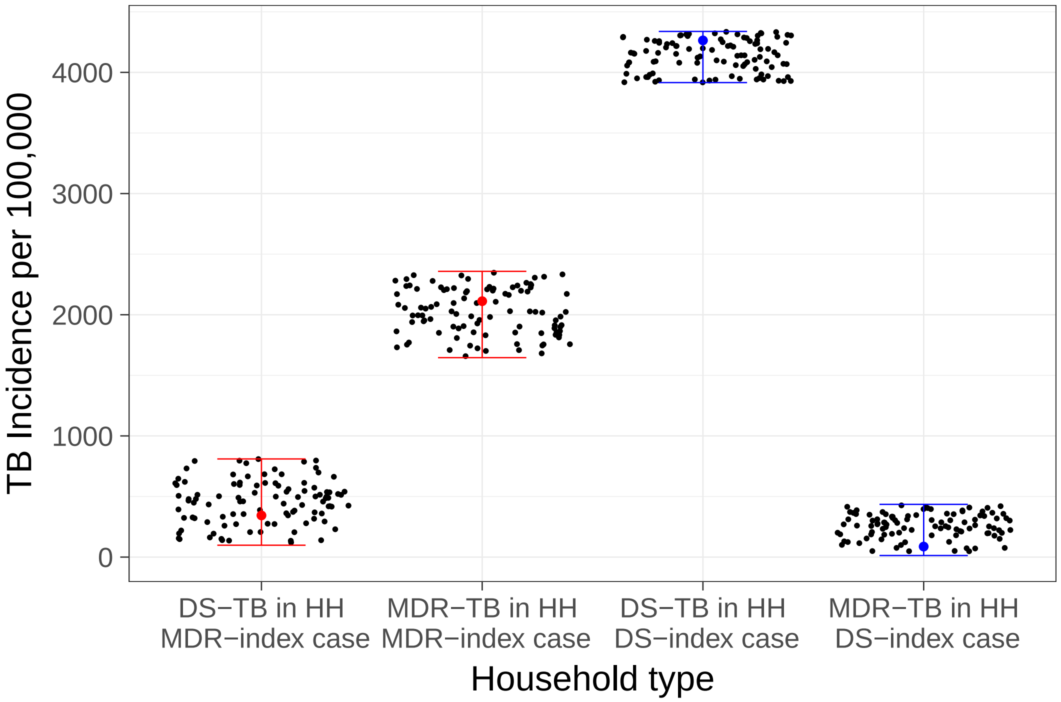

Figure 9: 100 example model fits. Black dots represent Model 1 output with scenario 1 parameters that matches to data shown in coloured ranges for each type of household (HH).

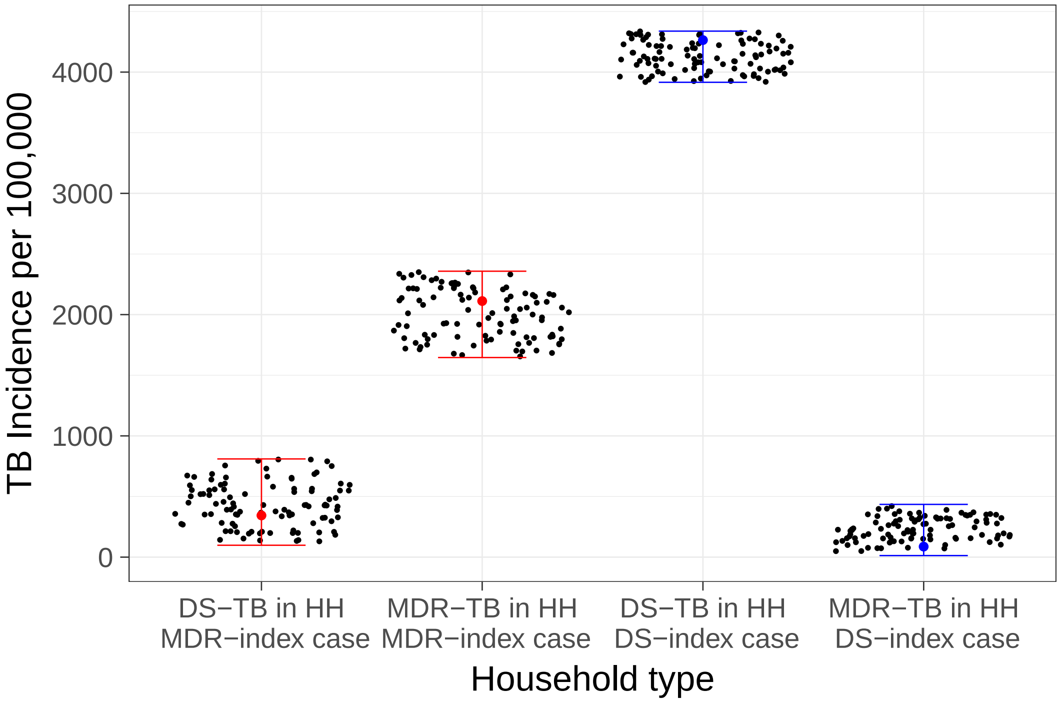

Figure 10: 100 example model fits. Black dots represent Model 1 output with scenario 2 parameters that matches to data shown in coloured ranges for each type of household (HH).

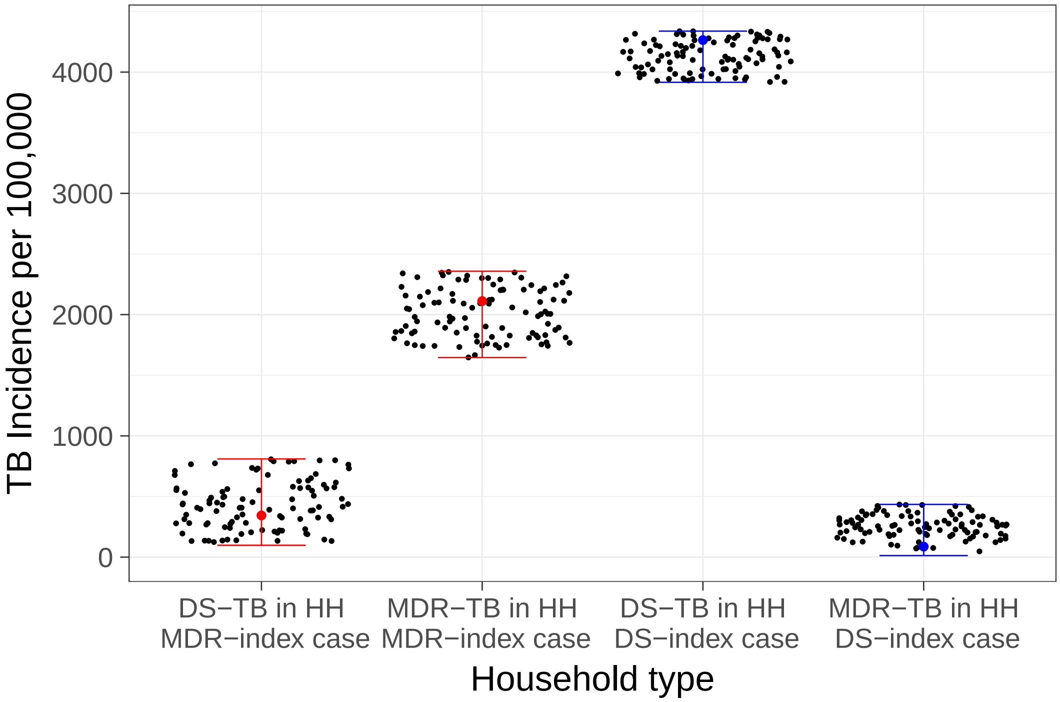

Figure 11: 100 example model fits. Black dots represent Model 1 output with scenario 3 parameters that matches to data shown in coloured ranges for each type of household (HH).

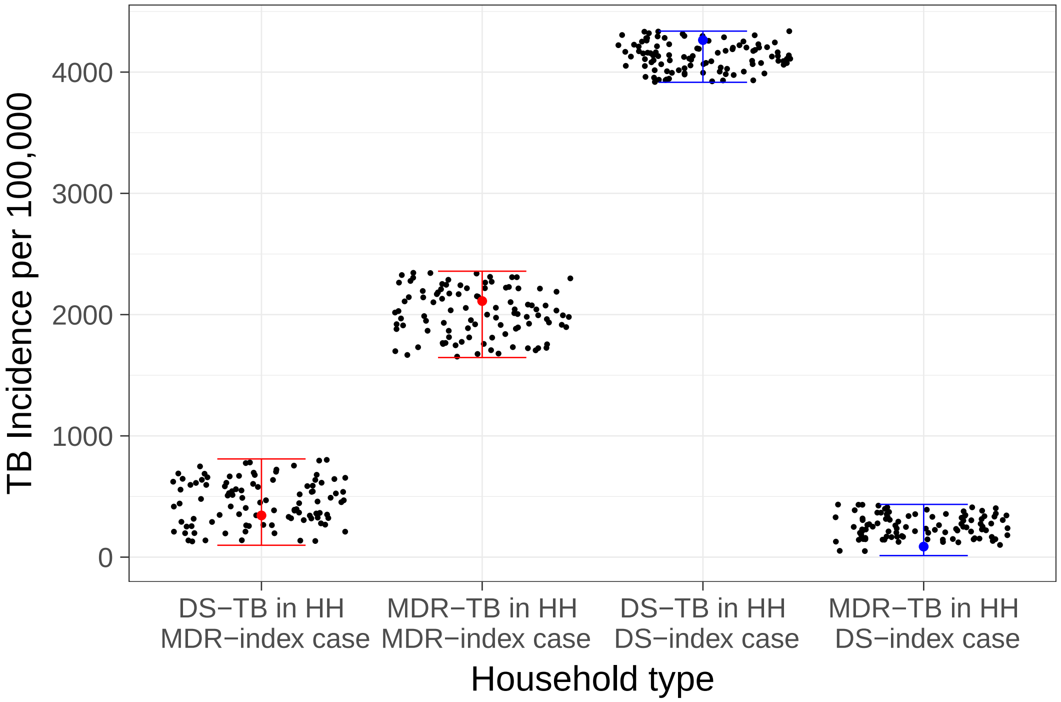

Figure 12: 100 example model fits. Black dots represent Model 1 output with scenario 4 parameters that matches to data shown in coloured ranges for each type of household (HH).

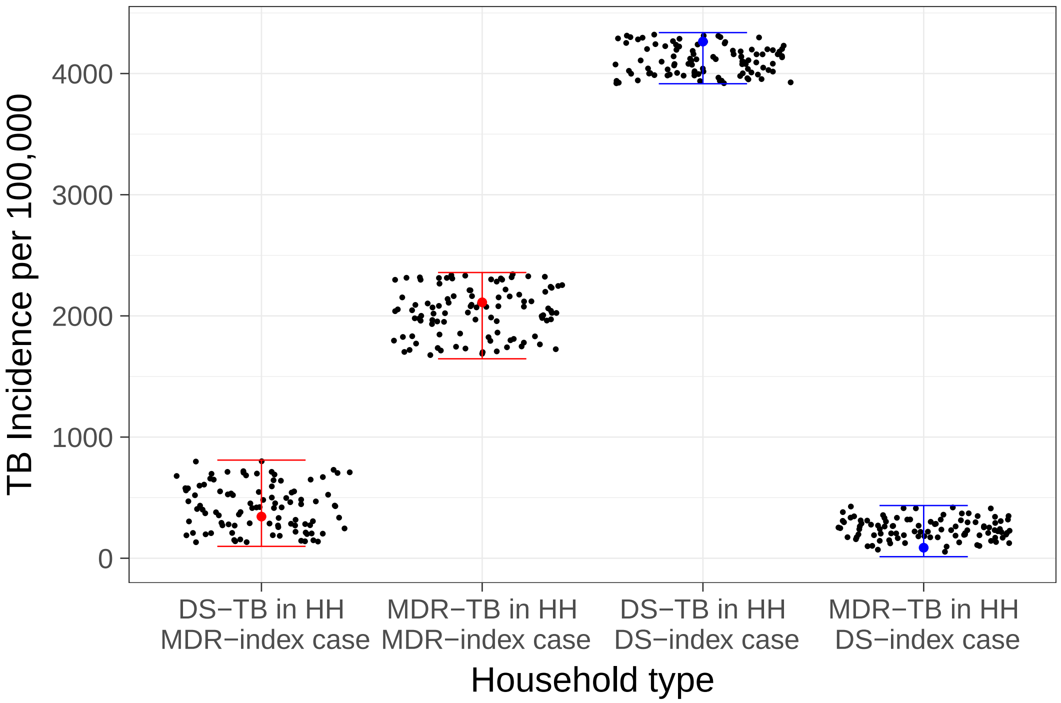

Figure 13: 100 example model fits. Black dots represent Model 1 output with scenario 5 parameters that matches to data shown in coloured ranges for each type of household (HH).

7 Trace and density plots for each unknown parameter for scenario analysis

The trace and density for each unknown parameter, from the three models are shown in Supplementary Figures 14-18.

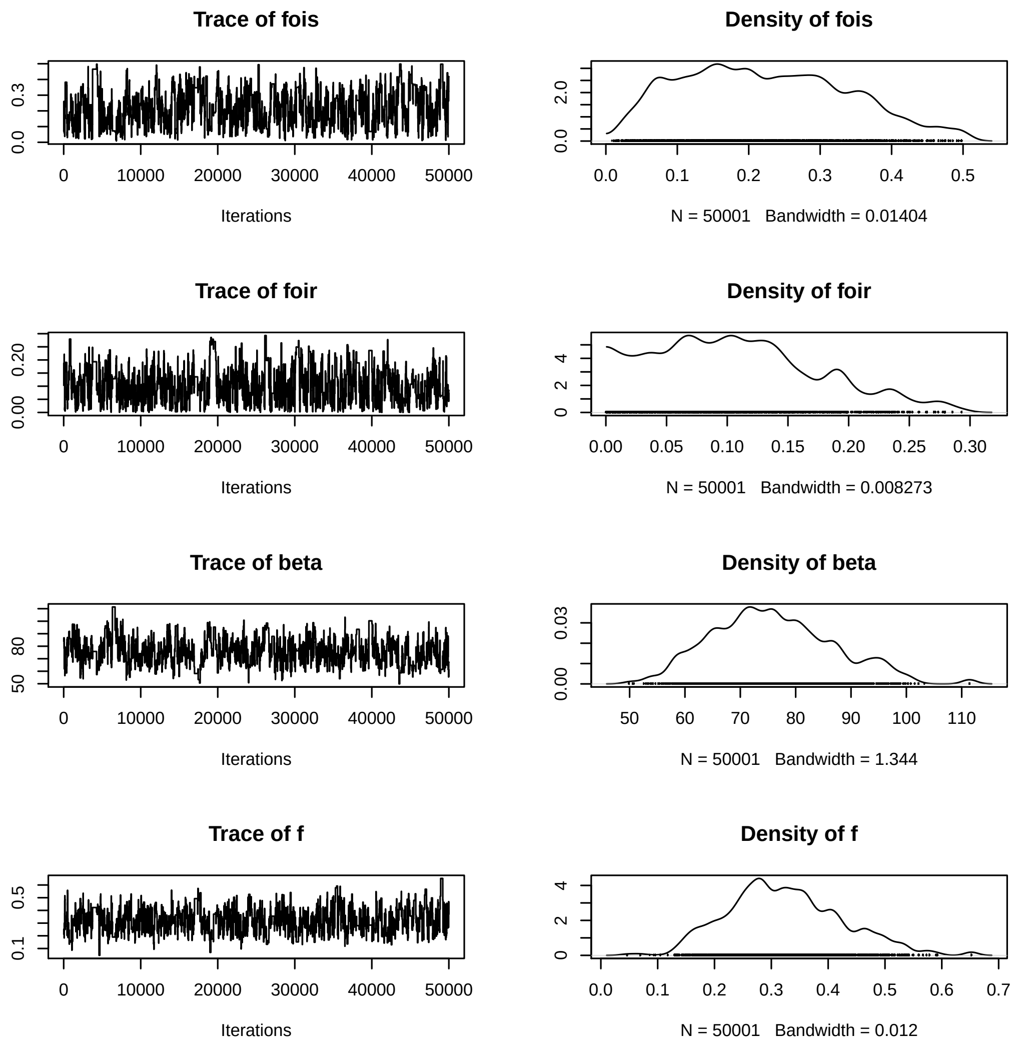

Figure 14: Trace and density plots for Model 1, scenario 1 (latent proportion)

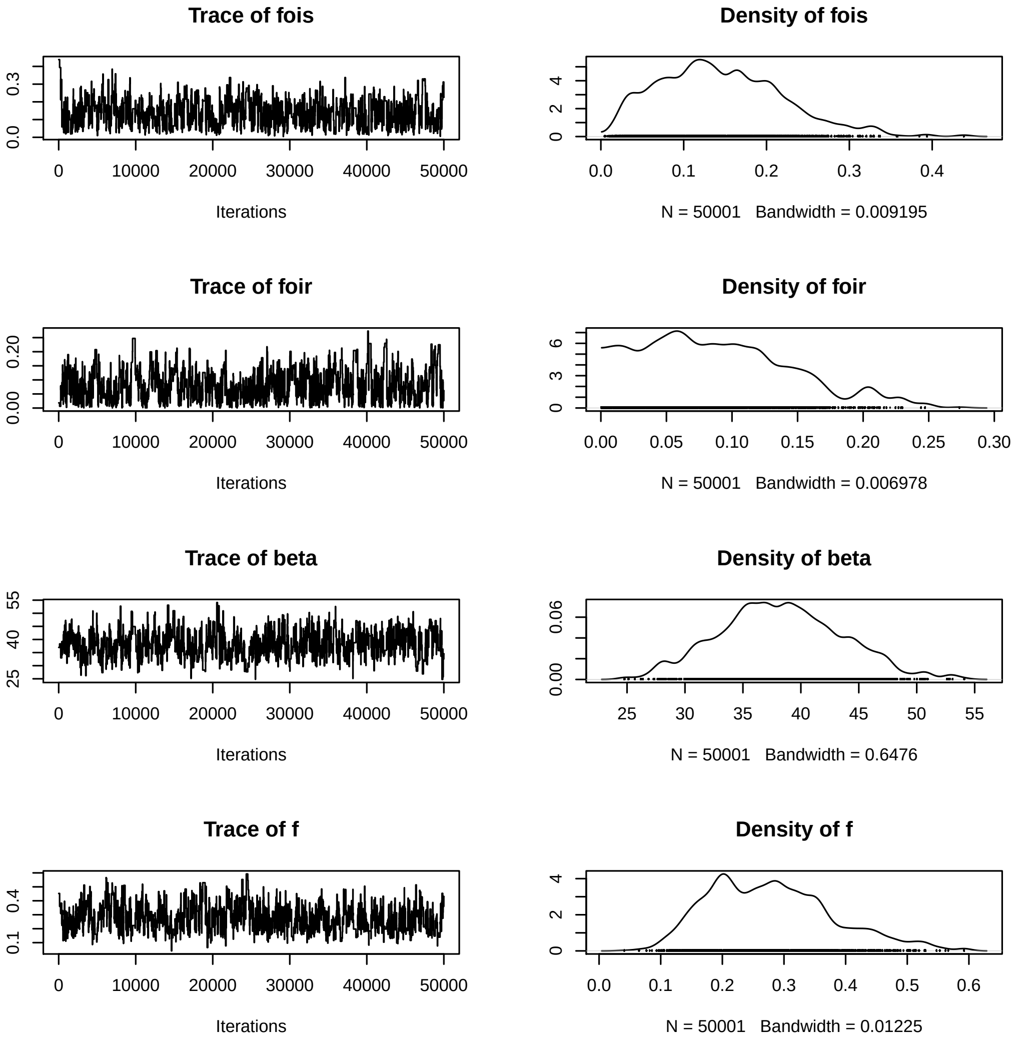

Figure 15: Trace and density plots for Model 1, scenario 2 (high TB incidence)

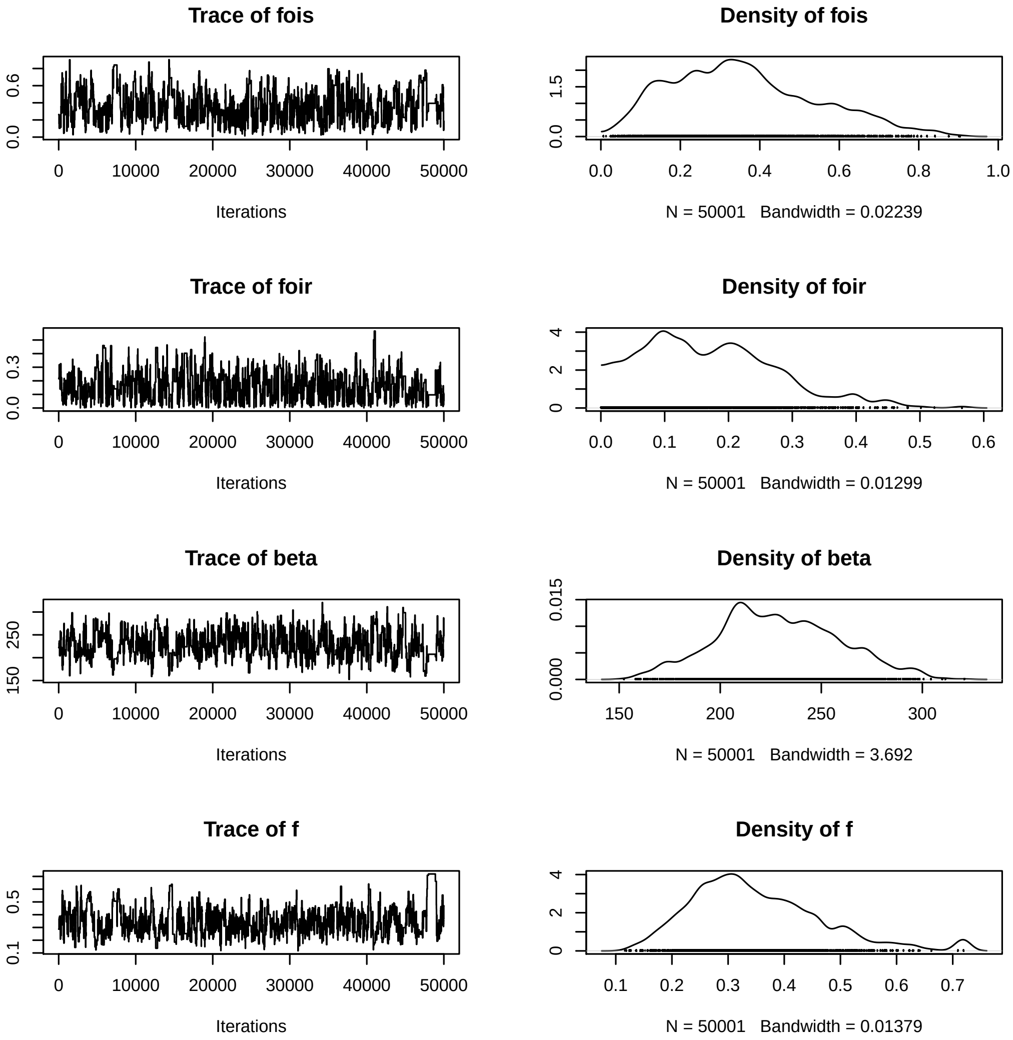

Figure 16: Trace and density plots for Model 1, scenario 3 (low TB incidence)

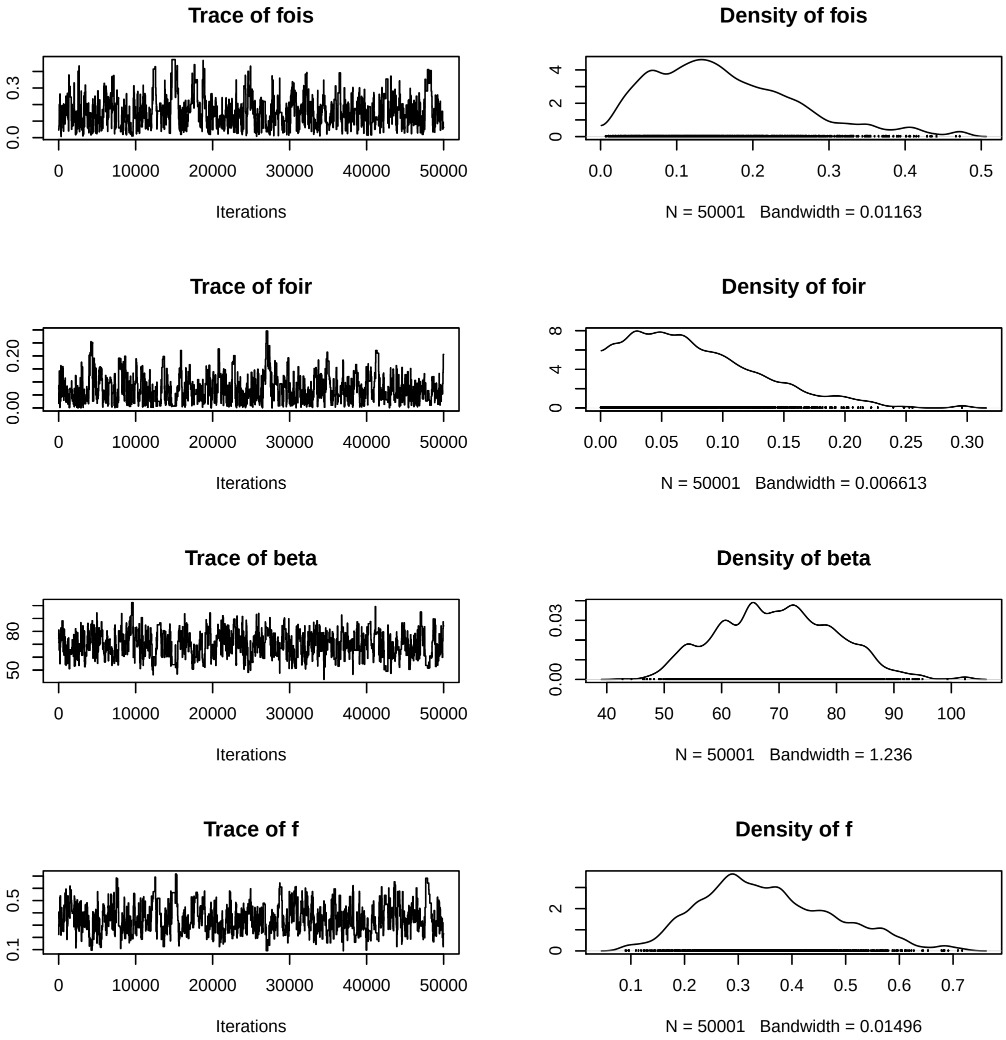

Figure 17: Trace and density plots for Model 1, scenario 4 (30 year burn in)

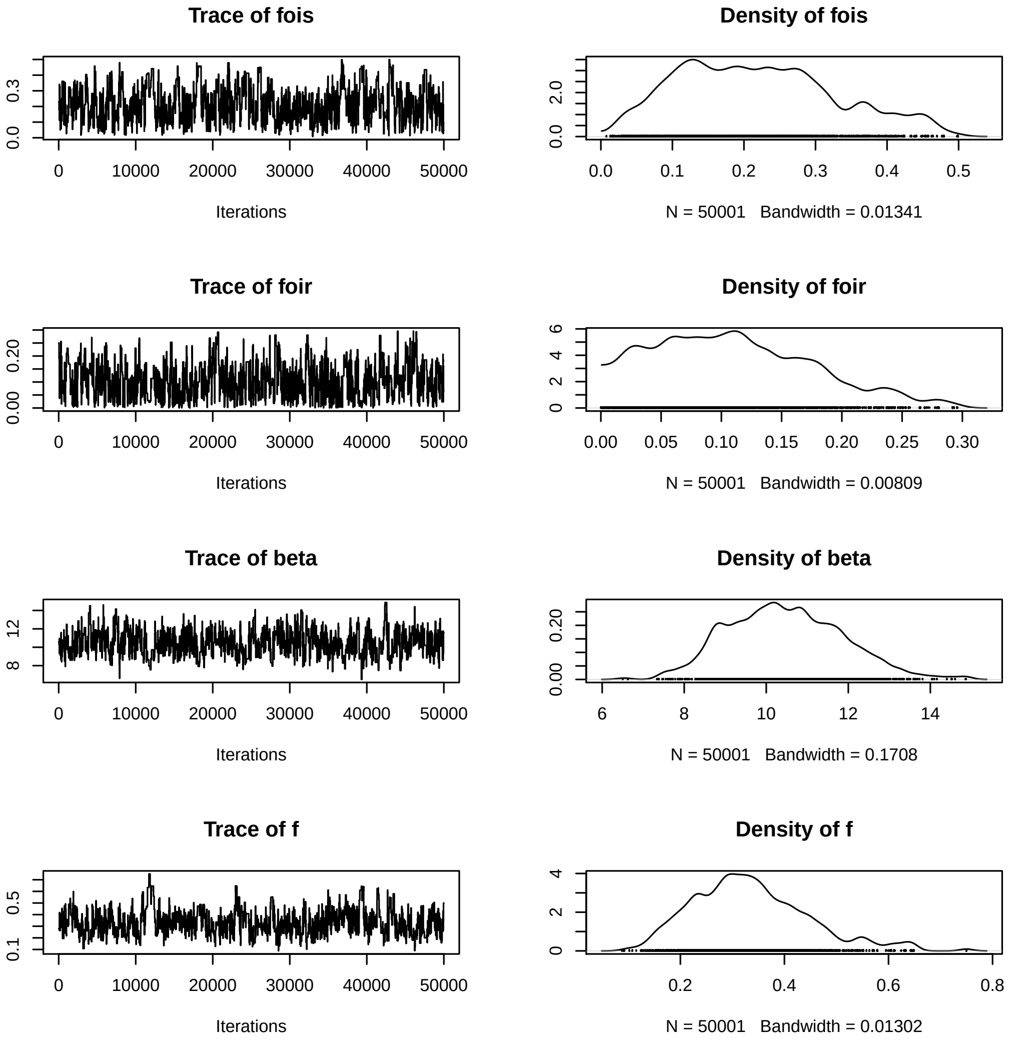

Figure 18: Trace and density plots for Model 1, scenario 5 (ho household saturation)

8 Scenario analysis results

The parameters estimates for the five scenarios are given in Table 1 and Figure 19.

Our first scenario analysis explored increasing the initial proportion of households that were initially infected with latent MDR-Mtb from 2% to 10% (in the pre-study). Fitting the four unknown parameters revealed that this increased MDR-Mtb latency proportion had very little impact on the estimates.

Our correlation analysis revealed four parameters (other than the four unknown parameters) to be correlated with TB incidence: the proportion of (re)infected individuals which progress to "latent fast" (p), the protection from developing active TB upon re-infection (χ), the proportion of new active cases which directly become infectious (d) and the progression rate of latent fast individuals to active disease (pf). The second scenario set these four parameters to be (p, χ, d, pf) = (0.25, 0.25, 0.75, 0.9) (high TB incidence) and the third (low TB incidence) to be (0.08,0.45,0.25,0.1). These second and third scenarios affected the estimates for the external force of infection and per capita transmission rate as would be expected due to the nature of the change in the natural history parameters. However, the estimates for the relative fitness (f) remain relatively consistent with our initial parameter set in Model 1 at approximately 0.30. Scenario 3 has a lower mean fitness at 0.22.

The fourth scenario, extended the initial run-in period from 10 to 30 years. All parameter estimates are similar to those of Model 1, including the relative fitness.

The fifth scenario removed the saturating household effect. The parameter estimates from this were also highly similar to the main analysis, except for the per capita transmission parameter, which was lower, reflecting the change to the model structure (no longer divided by household size).

**Scenario**	foi_s	foi_r	β	f
Model 1	$0.22(0.03-0.49)$	$0.10(0.01-0.26)$	$74.70(54.80-97.60)$	$0.32(0.15-0.62)$
1 (Greater proportion initially latently infected with MDR-TB)	$0.21(0.04-0.47)$	$0.10(0-0.26)$	$75.21(57.33-98.24)$	$0.32(0.15-0.54)$
2 (High TB incidence natural history parameters)	$0.14(0.03-0.32)$	$0.08(0-0.22)$	$38.1(28.22-48.96)$	$0.27(0.12-0.51)$
3 (Low TB incidence natural history parameters)	$0.34(0.07-0.76)$	$0.16(0.01-0.42)$	$227.41(171.21-292.05)$	$0.33(0.17-0.64)$
4 (30 years burn-in)	$0.15(0.03-0.40)$	$0.07(0-0.20)$	$69.44(51.52-89.46)$	$0.34(0.15-0.61)$
5 (No transmission saturation)	$0.21(0.04-0.45)$	$0.10(0-0.25)$	$10.4(7.95-13.3)$	$0.32(0.16-0.61)$

Table 1: Parameter estimates for the median and 95% credible intervals of the four unknown parameters from 50,000 MCMC iterations following a burn-in period of 10,000 iterations for the five scenarios explored within Model 1.

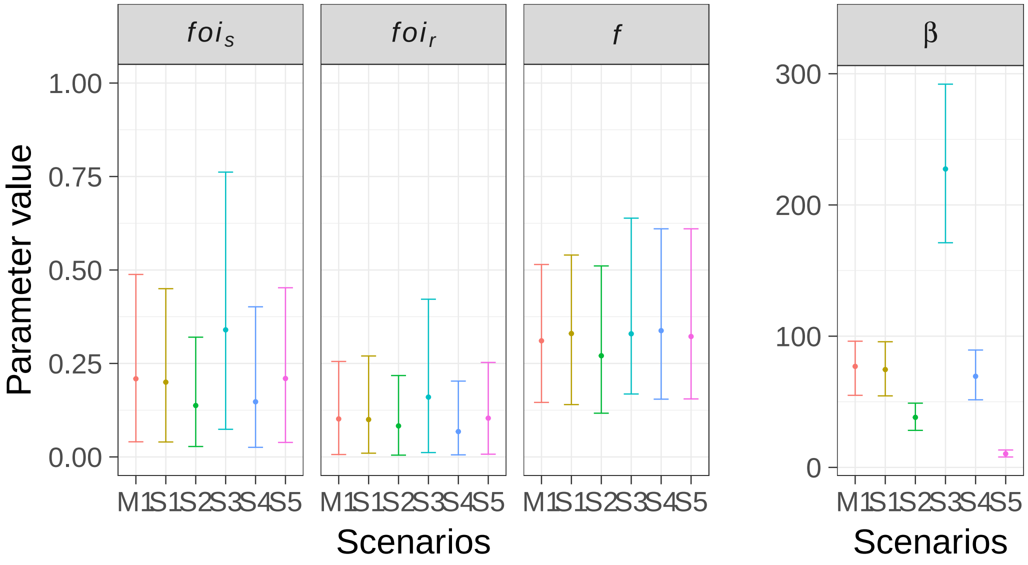

Figure 19: Fitted parameters for Model 1 and the five scenarios (S1-5). The units for the y-axis of the corresponding plots are: for the external forces of infection ('foi_s' and 'foi_r') proportion infected per year, for the relative fitness ('f') there are no units and for the per capita transmission rate ('beta') the units are effective contact rate per year.

9 Effective Reproduction Number estimates

The effective reproduction number (R) can be defined as the average number of secondary infections produced by an infected individual during the entire period of infectiousness. For our model, this can be approximated by taking the product of the transmission rate and the duration of infectiousness. This gives an approximate number of secondary cases generated by a single case of DS- or MDR-TB. The ratio of these two numbers (R_r for MDR-TB : R_s for DS-TB) provides another estimate of the impact of the resistance on MDR-TB transmission. In the pre-study period of our simulation, the ratio for Model *i* is:

$$\frac{R_r}{R_s} = \frac{\frac{\beta_s}{\omega_r(1-k_r)}}{\frac{f_i\beta_s}{\omega_s(1-k_s)}} = \frac{f_i\omega_s(1-k_s)}{\omega_r(1-k_r)} = f_i\frac{0.592}{0.384} = 1.54f_i \tag{1}$$

For our three models, inputting the estimates for f_i, gives ratios of approximately 0.49, 0.59 or 0.86. This suggests that MDR-TB has a substantially lower effective reproduction number than DS-TB in this setting, matching the results of the reduction in per capita transmission rate.

This calculation is only an approximation as it does not take into account the complexity of the latent states nor disease progression variation. We chose to use values from the pre-study period, as the case detection rate increased during the study.

References

[1] L. Grandjean, A. Crossa, R. Gilman, C. Herrera, C. Bonilla, O. Jave, J. Cabrera, L. Martin, A. Escombe and D. Moore, *The International Journal of Tuberculosis and Lung Disease*, 2011, **15**, 1164–1169.

[2] L. Martinez, A. Arman, N. Haveman, A. Lundgren, L. Cabrera, C. A. Evans, T. F. Pelly, M. Saito, D. Callacondo, R. Oberhelman *et al.*, *The American journal of tropical medicine and hygiene*, 2013, **89**, 507–515.
